# The Impact of Distance Learning on Parental Stress During the Second COVID-19 Lockdown in Germany

**DOI:** 10.1177/10664807221131011

**Published:** 2022-10-05

**Authors:** Isabelle May, Lena Hoerl

**Affiliations:** 1 Department of Educational Psychology and Research on Excellence, 9171 Friedrich-Alexander-Universitat Erlangen-Nurnberg, Erlangen, Germany

**Keywords:** COVID-19, stress, parents, resilience, homeschooling

## Abstract

Parental stress caused by monthlong school closures was measured as early as spring and summer of 2020. The present study investigated parental resilience during the second lockdown in Germany in January/February 2021 (n = 2,804). Based on an online questionnaire, parental stress, resilience, self-efficacy, children’s school abilities, and the perceived quality of lesson design in distance teaching were queried. Multiple linear regression analysis identified significant relationships between the experienced stress perception and the time spent supporting children in distance learning. We identified parental resilience as a predictor of stress experiences. It was possible to demonstrate the considerable influence of child variables and perceived lesson design on parental stress levels.

## Current state of research

The pandemic situation in December 2020 led to a second nationwide school closure. In Germany, schools remained closed from December 2020 to February 2021. The present study examines the impact of this second nationwide school lockdown on families in Germany. Data collected during the first lockdown in the spring of 2020 revealed an increased stress experience and dissatisfaction among many children and their parents (Porsch & Porsch, 2020; Ravens-Sieberer et al., 2021; Vodafone, 2020; Wildemann & Hosenfeld, 2020). The School Barometer research group identified influential factors that promoted distance learning in this period. Family emotions, the self-regulatory abilities of children, and the lesson design as perceived by adolescents and their parents played influential roles (Schwander et al., 2020). The design of distance learning had a significant effect on family satisfaction. Teachers’ willingness to communicate, regular contact between families and teachers, and meaningful feedback correlated with parents’ lower stress levels (Porsch & Porsch, 2020; Schwander et al., 2020). Further studies have identified parental requests for school support as better accessibility, more communication between teachers and students, and reliable feedback (Vodafone, 2020; Wildemann & Hosenfeld, 2020). Parental risk factors for an increased sense of stress, such as being a single parent, socioeconomic status, and belonging to occupational groups highly stressed by the crisis, were empirically determined (Bujard et al., 2020; Federal Ministry for Family Affairs et al., 2020). Unlike during the first crisis in the spring of 2020, governments were prepared to modify these stressful situations for affected families.

### Stress and influencing factors

As early as May 2020, empirical surveys showed the German general population’s overall reduced psychological well-being. Anxiety, stress, and depression increased (Fegert et al., 2020; Haas, 2020; Ravens-Sieberer et al., 2021). Several studies examining children’s well-being during the pandemic suggested significant negative consequences for their mental state (Fegert et al., 2020; Haas, 2020; Ravens-Sieberer et al., 2021).

According to Lazarus's transactional stress model, psychological stress arises from an individual’s assessment of the intensity of a stressful situation and knowledge of their coping options for that situation (Lazarus & Folkman, 1984). Most scientific articles have defined parental stress as a divergence between current parental role requirements and the perceived means available to parents to meet those requirements. If parents feel strongly stressed by their perceived expectations of responsibility in education and upbringing and care of their child due to immediate difficulties, a particular form of burden arises, which is parental stress or parental burden (Tröster, 2011). Based on Abidin’s modified parenting stress model, the distance learning situation is impacted by various stressors. The switch from face-to-face to distance learning challenged the adaptability of all affected schoolchildren. Abidin has found that children’s individual capability to adapt to new situations impacted on parental stress development.

The unique school closure situation and the resulting parental support in learning created a new parental role in children’s learning processes (Voss & Wittwer, 2020). To successfully cope with this new homeschooling situation, parents needed specific *parenting skills*. Parents may feel stressed if they perceive they are not doing justice to the distance learning task. *Personal limitations* could also induce parental burden. Experiences of personal limitations through the involvement in the upbringing and care of the child influenced stress sensations. The unique lockdown situation increased this personal limitation. *Lack of social support* caused parental stress. Restrictions on contact with grandparents and neighbors led to a significant reduction in social support for families.

In summary, some factors that are decisive for the development of parental stress were caused directly by the unique lockdown situation. In the following discussion, we briefly present the influencing factors we examined.

#### Time

The time required to educate may affect parental burden. During spring 2020, German parents supported their children in distance learning for an average of 3 h a day (Federal Ministry for Family Affairs et al., 2020; Vodafone, 2020). Some parents perceived this expenditure of time as a burden. Most of the parents surveyed predicted such a permanent expenditure over a more extended period as a stressor for their future (Vodafone, 2020). Politicians responded by successively expanding emergency care to more occupational groups and families with risk factors. Another political reaction was the increase in children’s sick days for families with two parents from 10 to 30 days per parent and child and for single parents to 60 days (Federal Ministry for Family Affairs et al., 2021). A study by Barmer Health Insurance has shown that in the first quarter of 2021, insured persons took 37% more children’s sick days than in the first quarter of 2019 and 2020 (Barmer, 2021). With these political modifications, the amount of parental support time varied. This modification suggests that the research question of whether the time the parents spent supporting distance learning influenced parental stress growth.

#### Resilience

Resilience defines a person’s psychological robustness in stressful life situations. Resilience is the competence to master challenging moments without consequential damage (Diers, 2015). The COVID-19 pandemic and its consequences posed a challenge for the population. Families with school-age children were confronted with multiple problems. The families were deprived of important resilience-promoting protective factors, such as other caregivers (grandparents, relatives, neighbors, and teachers). According to Werner, the number of supportive helpers in the family from early childhood to the age of 10 is one of the most critical factors in predicting a positively adapted, age-appropriate development (Werner, 2011). Families had to deal with health and financial concerns during the weeklong school closures. The studies from spring 2020 only show a partially uniform picture. Some parents reported positive emotions while distance learning (Porsch & Porsch, 2020). Most studies identified mostly stressed parents, but they also showed a minority of parents with little or no burden (Bujard et al., 2020; Federal Ministry for Family Affairs et al., 2021; Vodafone, 2020).

#### Competencies

An adequate *adaptation* to the changed school situation required specific child competencies. The effectiveness of children’s competencies in distance learning impacted on parental stress. In the spring of 2020, children were coping differently with the COVID-19 school situation. Gruber distributed adolescents into two groups in terms of their academic competencies and the situation:

Some find it good to learn at their own pace and rhythm. To work in a more self-determined way, they now learn more effectively, according to their statements, and cope well with the situation. Others have problems, e.g., concerning the structuring of their day, tasks, and motivation. There are significant differences in daily learning time. (Schwander et al., 2020, p. 8)

Consequently, school skills, such as organizational competence and maintaining motivation and attention play a role in dealing with family stress. A child's motivation, self-organization, and concentration ability are essential to school success during face-to-face teaching. This survey aims to demonstrate the influence of these competencies during school closures on the child’s learning process and family stress.

#### Teaching

Factors that increased the burden included *doubts about one’s educational competence*. The perceived distance-learning design could significantly influence parents’ perceived educational competence. Communication frequency and teacher feedback were mentioned as significant quality features (Bujard et al., 2020; Porsch & Porsch, 2020; Schwander et al., 2020; Wildemann & Hosenfeld, 2020).

#### Question

The present study explored the impact of distance learning on parental stress during the second COVID-19 lockdown in Germany. To specify this issue, we aimed to answer the following research questions:

H1: Did parents feel stressed during the school closure of the second COVID-19 wave? (Stress)

H2: Did the time spent supporting children’s distance learning affect parents’ sense of stress? (Time)

H3: Did resilience competencies influence stress perception? (Resilience)

H4: Did children’s school skills influence their parents’ perception of stress? (Competencies)

H5: Did perceived lesson design influence parental stress? (Lessons)

H6: Did a child’s age influence parental stress? (Age)

## Data and Methods

### Participants

From January 16 to February 22, 20201, an online questionnaire (www.umfrageonline.com, enuvo GmbH, Zurich, Switzerland) was distributed by email via the parents’ associations and social networks. Participation was voluntary. A total of 3,472 people participated, of whom 3,066 completed the questionnaire. After correcting for incorrect answers, the total number of participants was 2,804 (n = 2,804) parents. Demographic data of the sample can be found in Table 1.

**Table 1.** Descriptive Data.

		n	%
Gender	Male	365	13
	Female	2,439	87
Age (years)	18–30	196	7
	31–40	1,372	49
	41–50	1,092	39
	>51	140	5
Education	No education	84	3
	Secondary school	532	19
	Associated degree	756	27
	Bachelor's degree	364	13
	Master's degree/doctoral degree	1,064	38
Working hours	Not working	280	10
	Part time up to 50	700	25
	50 or more	1,120	40
	Full time	700	25
Work situation	100% home office	1,176	42
	Switching from home office to in-person	756	27
	100% in-person	868	31
Number of children	1	999	36
	2	1,322	47
	3	365	13
	4	118	4
Family situation	Single parent	476	17
	Divorced in an alternate model	140	5
	Two-parent family	2,184	78

### Survey

The questionnaire consisted of 39 questions on demographic data (n = 9) and on the current family situation (n = 30, Appendix 1). The latter were structured in items to:

- H1: Stress (n = 10);
- H2: Time (n = 4);
- H3: Resilience (n = 6);
- H4: Competencies (n = 4); and
- H5: Lessons (n = 6).

The participants were able to answer the questionnaire in English or German.

#### H1: Stress

Ten questions raised the stress situation. Three questions measured the general feeling of stress. Four questions specified the feeling of stress triggered by distance learning. These four questions were adapted from the parenting stress questionnaire by Abidin in the German version of Tröster. Parental concerns were raised through three further questions adapted to Abidin (Tröster, 2011). The answer option was a 4-point Likert scale (4 = fully true, 3 = partially true, 2 = rather not true, 1 = does not apply). Table 2 presents the questions and their good Cronbach’s alphas.

**Table 2.** Stress Items.

1	The current general situation (COVID-19 restrictions, financial situation, health concerns) is weighing heavily on me right now	*0.84*
2	I feel stressed and overwhelmed by my child's support in distance learning	
3	Supporting my child in distance learning is mainly responsible for my current stress situation	
19	During homeschooling, I feel stressed and overwhelmed when I help my child…	*0.75*
19a	To motivate themself	
19b	Plan the lesson day	
19c	Fix technical problems	
19e	Understand work orders and content	
20	I am concerned that my child will be affected by the school closure through…	*0.80*
20a	Knowledge gaps	
20b	Reduced social skills	
20c	Losing the motivation to learn	

*Note*. R^2^ = 0.42 (n = 2,804, *p* <.001). CI = confidence interval for B.

#### H2: Time

Time spent was raised via four questions. The first question: “*I have enough time to support my child in homeschooling*,” the parents answered via the above 4-point Likert scale. The participants indicated the quantitative time required to support the children in distance learning in hourly increments of 1–10 h per day. The extent to which the time spent restricts the exercise of the work and leisure behavior of the parents was asked via two questions, which could be answered with the previously mentioned 4-point Likert scale.

#### H3: Resilience

Resilience-enhancing factors were measured with six questions and answered via the 4-point Likert scale. The first question related to the self-concept of parental ability. Parents indicated whether they could sufficiently support their child in distance learning. The following two questions outlined the general stress management strategies, knowledge of dealing with stress, and the competence to gain appropriate help. The following statement of the item group “resilience” titled: “*We make the most of distance learning—especially when it is difficult*,” reflects the respondents’ optimism. Most recently, parents reported whether they usually found a way out of difficult situations. The last two questions were based on the resilience scale (Leppert et al., 2008). The Cronbach’s alpha of the six questions indicated acceptable reliability with α = 0.70.

#### H4: Competencies

Parents’ estimates assessed children’s academic abilities. For each schoolchild, we offered a separate block of questions. Besides the child's age and the time required to work on the school tasks daily, a question about children's self-regulatory learning skills was asked. The exact wording, as well as the respective competencies, can be found in Table 3.

**Table 3.** Text of the Items on Pupil Competences.

Competence	Item
Organization	*My child can organize their learning well independently. (Breaks, structuring of the daily routine)*
Motivation	*My child can motivate themself well to cope with the tasks of distance learning*
Concentration	*My child concentrates on their work*

#### H5: Perceived lesson design

Six questions about the perceived quality of lesson design consisted of two thematic blocks of three questions each. The first block described the communication between teachers and children, parents and teachers, and the frequency of correction by teachers. The parents were asked how often their child communicated weekly with the teacher via email, video chat, and telephone. The parents could also provide the same information via their communication channels with the teachers. The following answer options were provided: 1 = not at all, 2 = one time, 3 = two times, 4 = three times, 5 = four times, 6 = five times, 7 = more than five times. The second block of questions related to the design of distance learning. First, it was recorded how often the teachers corrected the tasks set. The next question was about the digital lessons: “*Digital solutions for distance learning are optimally implemented (learning platform, explanatory videos, sufficient video conferences)*.” Afterward, it was asked whether distance learning was optimally designed. This question also related, in particular, to the analogous design of lessons. Finally, parents were asked to assess the extent to which the work assignment designs were self-explanatory for their children. These questions were, again, answered via a 4-point Likert scale.

#### Statistical Strategy

All analyses were performed using R Statistical Software (v4.1.2; R Core Team, 2021). To evaluate the distribution of ordinal-scaled responses, nonparametric χ² tests were performed. The distribution of most items was not expected. The descriptive statistics were presented as percentages, median, mean, and *SD*.

#### Data analysis

To test internal consistency, we calculated the Cronbach’s alphas for items with several questions. To learn more about the relationships within the stress items, correlations were calculated. Table 4 illustrates descriptive statistics for stress, time, resilience, organization, motivation, concentration, perceived lesson design, and children’s ages. We also calculated a Cronbach’s alpha for the variable school skills consisting of organization, motivation, and concentration items. Since this was only 0.40, these items could not be combined into a variable. Instead, we used these three as three standalone variables.

**Table 4.** Regression Analysis Summary for Variables Predicting Parental Stress.

Variable	B	β	t	*p*
Stress	5.30		58.60	<.001
Time	0.12	0.26	17.48	<.001
Resilience	−0.62	−0.41	−26.53	<.001
Organization	−0.01	−0.02	−1.41	.159
Motivation	−0.01	−0.02	−1.16	.248
Concentration	−0.08	−0.12	−7.65	<.001
Lesson design	−0.18	−0.15	−9.68	<.001
Child's age	−0.03	−0.12	−7.83	<.001

#### Inference statistics

The hypotheses referred to the influence of several metrically scaled variables (time, resilience, organization, motivation, concentration, and perceived lesson design) on a metrically scaled dependent variable (stress). The appropriate analysis method for this situation was multiple linear regression analysis. Before applying multiple linear regressions, the prerequisites of this method, normal distribution of residuals, homoscedasticity, absence of multicollinearity, absence of autocorrelation, and linear relationships between the dependent and independent variables were checked, and all conditions were fulfilled.

## Results

### H1: Did parents feel stressed during the school closure of the second COVID-19 wave?

All stress items correlated strongly to moderately with each other. The first three stress items already had an excellent Cronbach’s alpha, correlated strongly. Parents who felt stressed by the children's motivation also found distance learning a significant stressor. The more support in planning the lesson day and technical support to be provided to the parents, the higher their generally stated stress level was. The more stressed the parents felt, the more worried they were about their children. The large correlation between the stress to help motivate the children and the worry about their loss of motivation was significant (r = .62, *p* <.001). The more stressed the parents felt about distance learning support, the more concerns about children’s knowledge, social competence, and motivation were measured.

### H2–H6: Time, Resilience, Competence, and Lesson Design

**Figure 1.**
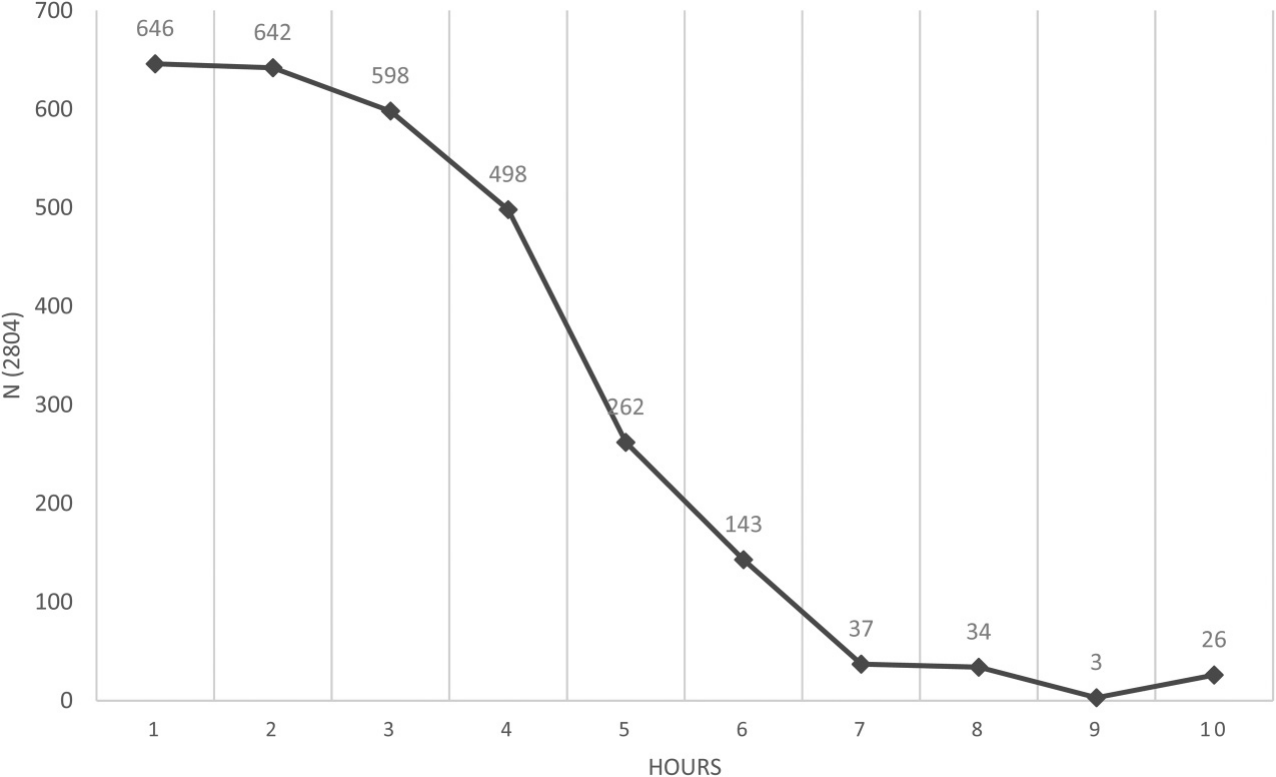
Time. How much time do you spend each day on helping your children?

The majority (64%, χ^2^ (3, n = 2,804) = 573.56, *p* < .001*, M = 2.26; Mdn = 2, *SD* 1.75, Likert 1 and 2) of the parents reported not having enough time for the appropriate support of their own children in distance learning. Figure 1 shows that the daily parental time spent supporting the children. On average, it was 3h a day (χ^2^ (9, n = 2,804) = 2397.9, *p* < .001*, M = 3.01, Mdn = 3, *SD* = 1.75). More than half of the parents felt the impact of supporting distance learning on the usual practice of their profession (Likert 3 and 4 = 64%, χ^2^ (3, N = 2,804) = 232.32, *p* <.001*, M = 2.79, Mdn = 3, *SD* = 1.08) and in the context of their leisure activities (Likert 3 and 4 = 65%, χ^2^ (3, n = 2,804) = 399.98, *p* <.001*, M = 2.92, Mdn = 3, *SD* = 1.02). The children spent an average of 3h a day doing schoolwork (M = 4.19, Mdn = 4, *SD* = 1.83).

The regression model shows an F-value of F (6; 1,511) = 180.60, *p* <.001. Since the *p*-value of the F-test was <.05, the regression model had a significant explanatory quality. The adjusted R^2^ of the regression results were R^2^ = 0.42. Table 4 presents the coefficient block of the regression. Regarding the regression table (Table 4) the following statements according to the hypothesis can be made:

#### H2: Did the time spent supporting children’s distance learning affect parents’ sense of stress?

A *p*-value of <.05 showed that the time required significantly affected stress.

The positive regression coefficient B indicated that stress proliferated significantly with increasing time expenditure.

#### H3: Did resilience competencies influence stress perception?

Resilience had a *p*-value below .05 and a negative regression coefficient. Thus, resilience had a significant negative effect on stress. The higher the resilience, the lower is the parental stress measured.

#### H4: Did children’s school skills influence their parents’ perception of stress?

The variable organization and motivation both had no significant effect on stress. Concentration had a significant negative effect on the dependent variable stress. The higher the concentration, the lower is the parental stress level.

#### H5: Did perceived lesson design influence parental stress?

Lesson design had a significant negative effect on stress. The better the perceived quality of the lesson design, the lower is the parental stress level measured.

The value of the standardized regression coefficient β illustrated which variables examined had the most decisive influence on stress. Table 4 shows that β is the largest in terms of the amount for the variable resilience, so this model demonstrates that resilience had the most substantial influence on stress, followed by time expenditure and lesson design.

#### H6: Did a child’s age influence parental stress? (age)

A remarkable negative effect existed in relation to a child’s age. The older the children were, the less stress was perceived by their parents.

## Discussion

Most parents rated their stress levels as high during the second school closure in this study. The measured values corresponded to the stress levels collected during the first school closures in the spring of 2020 (Porsch & Porsch, 2020; Wildemann & Hosenfeld, 2020). In our questionnaire, most parents stated that the general COVID-19 situation weighed heavily on them. Therefore, the school situation should not be regarded as the sole stressor, but it still plays a decisive role. The more precisely the role of distance learning in connection with negative emotions was asked, the more the proportion of heavily burdened parents decreased.

Nevertheless, most parents believed that the school situation was mainly responsible for their perceived stress. Empirical research results from the first lockdown in the spring of 2020 were thus replicated for the winter of 2021 (Porsch & Porsch, 2020; Ravens-Sieberer et al., 2021; Vodafone, 2020; Wildemann & Hosenfeld, 2020). Although there were policy changes in the design of working conditions, emergency school care, contact restrictions for children and families, and lesson design, no significant changes in the stress situation was measured.

The time factor played a decisive role during the second school closure. Parents who felt that they did not have enough time to care for their children felt more burdened. On average, parents spent as much time schooling their children as they did in the spring of 2020 during the first school closure (Bujard et al., 2020; Vodafone, 2020; Wildemann & Hosenfeld, 2020). One could conclude that the political measures (children’s sick pay and guidelines for the design of lessons) had little influence on parental stress. No significant correlation between the time factor and the stress prevention items (resilience) could be found. Parents who invested significant time supporting their children felt more stressed but showed just as much confidence and self-efficacy as parents who spent less time. The proportion of children’s sick days increased slightly, and there was, in principle, the same right to these children's sick days for all employes. This option was probably not fully used due to the operational situation, the order situation, fear of dismissals, and the option of the home office to relieve working parents sufficiently.

Unlike previous studies on parental stress during the COVID-19 pandemic, our research surveyed perceived student competencies, such as motivation, concentration, and organization, as well as the intensity of parental support in these areas.

The individual *adaptability* of the child had a direct influence on the stress experience of the parent(s). Thus, parents who strongly supported their children in motivating and structuring felt more stressed than parents who felt helpless in this area. Participants who said that their children could not concentrate particularly well felt a higher burden when supporting distance learning. Promoting the motivation to learn and cope with the tasks was challenging for most parents. Most parents felt burdened by motivating the children. The children’s self-regulatory abilities, such as the motivation to cope with the tasks set by the teachers and the ability to concentrate, influenced the parental stress perception.

Children with poorly trained self-regulatory abilities could hope for less school success during distance learning and thus had to reckon with more burdened parents. This created a negative cycle because the stressed parents negatively influenced the children’s emotional state, which in turn affected the ability to learn. The fact that children with poorly trained self-regulatory abilities showed poorer school performance also corresponded to the situation of these children in mainstream school. Concentration and motivation are essential determinants of school performance (Schrader & Helmke, 2008). Since the teachers and classmates are usual possible sources of motivation, structuring aids, or concentration frameworks, parents had to absorb these tasks and found themselves in a stressful situation. The gap between weak and strong students could thus be widened due to school closures.

In Abidin’s parenting stress model, an important role is attributed to the child’s attention span (*adaptability*) as well as the belief *(doubts)* in *parenting courage* (Tröster, 2011). Both are stressors, and this study correlated them significantly with all stress items.

Looking at the connections between the lesson design and the parental stress level, effective design of the distance learning environment could positively affect the family climate. A well-structured and motivational learning environment could promote school success and influence the mood of the students and their parents. Parents who rated lesson design by their children’s teachers as adequately felt less burdened by distance learning. Thus, the distance learning education standards issued by the Ministers of Education and Cultural Affairs of the Federal States for the design of lessons in their consistent implementation can contribute in reducing parental stress experiences.

## Limitations

The following limitations should be considered when interpreting our results. First, the questionnaire distribution was random. The sample was not representative. Second, stressed parents certainly had a stronger motivation to fill out this questionnaire. Extremely stressed parents, in turn, may have had trouble finding time to complete the survey. Third, since the survey was distributed via the internet, families without a digital device could not participate. Although we offered an English version, the linguistic diversity of immigrants living in Germany was not covered. Fourth, the children's feelings, worries, and competencies were not judged directly but only by the parents’ impressions. Fifth, the lesson design was measured by parental perception. The findings cannot be transferred to other countries or educational institutions.

## Further considerations

Even during the second school closure, most parents said they felt stressed by the burden of supporting their children’s distance learning. The improvements and protective measures of the policy could not reduce the increased stress experienced by the parents. However, most parents said they wanted to make the most of distance learning—especially when it was difficult. The study identified factors that promoted this resilience and reduced stress levels.

Children’s motivation, concentration, and an optimally designed learning environment influenced parental stress experience, and the required support time. In addition to optimal lesson design by teachers, different framework conditions should be created to make distance learning easier for families in the future. To minimize the time required from parents, consideration should be given to enable an early offer of work in small groups in compliance with hygiene regulations at school. A regular meeting would relieve the parents in motivating, structuring, and explaining the tasks. The involvement of teacher training students as digital or natural learning facilitators could have a supportive effect. These measures would reduce the need for individual support by parents. A reduction in the curricular content of all grades and their official announcement by the Ministers of Education and Cultural Affairs via the media would undoubtedly have reduced parental concerns about the development of knowledge gaps. As age had an impact on parental stress, primary schools should implement self-regulated learning lessons from the first class.

Future research should focus on motivational measures in the context of distance learning, as well as programs that strengthen parental resilience. In addition, vulnerable groups (children with learning difficulties, children with attention-deficit hyperactivity disorder, parents in systemically important professions, single parents, and parents with many children) should be monitored and supported. The status quo of primary schools self-regulated learning competencies should be examined, self-regulated trainings should be offered and evaluated.

## Appendix 1. Questionnaire

**Table:**

Question
The current general situation is weighing heavily on me right now. I feel stressed and overwhelmed by my child's support in distance learning. Supporting my child in distance learning is mainly responsible for my current stress situation. I have plenty of time to support my child in homeschooling How much time do you spend each day helping your children homeschool? The time spent caring for my child at homeschooling limits The normal execution of my profession My free time I think that I can support my child sufficiently in distance learning I know how to counteract stress in myself If I feel overwhelmed with my child's support, I get help We make the most of distance learning—especially when it's difficult When I'm in a difficult situation, I usually find a way out The following information applies to your children Age Time Organization Motivation Concentration My child communicates with the teacher every week I communicate with the teacher every week The teacher corrects the tasks from homeschooling Digital solutions for distance learning are optimally implemented I think that the teacher optimally designs distance learning The work orders are self-explanatory for my child Stress Concern State Family situation Sex
